# Higher betaine is associated with lower incidence of microvascular complications in type 2 diabetes (Zodiac‐61)

**DOI:** 10.1111/eci.13873

**Published:** 2022-09-30

**Authors:** Amarens van der Vaart, Martin H. de Borst, Stephan J. L. Bakker, Margery A. Connelly, Peter R. van Dijk, Robin P. F. Dullaart

**Affiliations:** ^1^ Department of Internal Medicine, Division of Nephrology, University Medical Center Groningen University of Groningen Groningen The Netherlands; ^2^ Department of Internal Medicine, Division of Endocrinology, University Medical Center Groningen University of Groningen Groningen The Netherlands; ^3^ Laboratory Corporation of America® Holdings (Labcorp) Morrisville North Carolina USA

**Keywords:** betaine, microvascular complications, type 2 diabetes

**Abbreviations**
NMR: Nuclear magnetic resonance
T2D: Type 2 diabetes
SAM: S‐adenosylmethionine
VEGF: Vascular endothelial growth factor
ZODIAC: Zwolle Outpatient Diabetes project Integrating Available Care

*To the editor:*

Betaine (N,N,N‐trimethylglycine) is a gut microbiome‐derived osmolyte, and its concentration in plasma is in part dependent on dietary intake.^1^ Dietary sources include shellfish, wheat, beets and spinach. Betaine serves as a methyl donor in a reaction catalysed by betaine homocysteine methyltransferase, which converts homocysteine to methionine.^1^ Betaine is thus required for the remethylation of homocysteine to methionine, a precursor of the universal methyl donor S‐adenosylmethionine (SAM), with an essential role in many body processes. Betaine is inversely associated with adiposity, hypertension and elevated plasma triacylglycerols.^2^ Low plasma betaine levels may also predict the future development of type 2 diabetes (T2D).^3^, ^4^ In a mouse model of oxygen‐induced retinopathy, betaine decreased neovascular tuft formation.^5^ Betaine also inhibited vascular endothelial growth factor (VEGF)‐induced proliferation in retinal microvascular endothelial cells.^5^ In another study, betaine was found to inhibit VEGF expression in a rat retina model of streptozotocin‐induced diabetes.^6^ Moreover, betaine attenuates proliferation of mesangial cells cultured under high glucose conditions,^7^ whereas low levels of SAM were associated with DNA demethylation in peripheral nerves of mice with diabetic neuropathy.^8^ Given these findings, and in the absence of data on the role of betaine in humans, we tested the hypothesis that betaine is associated with the development of microvascular complications in T2D.

We studied the association between plasma betaine and microvascular complications in the Zwolle Outpatient Diabetes project Integrating Available Care (ZODIAC) study, a Dutch cohort of individuals with T2D, which has been described elsewhere.^9^ Analyses were performed in participants with available plasma samples at baseline and data on microvascular complications at baseline and during follow‐up. We excluded participants with microvascular complications at baseline. Microvascular complications were defined as the presence of either retinopathy, nephropathy or neuropathy. Diabetic retinopathy was investigated by fundus photographs obtained with a retinal camera and judged by an ophthalmologist. Nephropathy was defined as at least two repeated occurrences of albuminuria (albumin‐to‐creatinine ratio >3.5 mg/mmol in women and >2.5 mg/mmol in men), or one occurrence if the patient used an angiotensin‐converting enzyme (ACE)‐inhibitor or angiotensin receptor blocker. Neuropathy was defined as two or more errors out of three tests of foot sensibility, using a 5.07 Semmes‐Weinstein monofilament, at least at one foot. Betaine levels were assessed in EDTA‐anticoagulated plasma samples using nuclear magnetic resonance (NMR) spectrometry (LabCorp, Morrisville, NC) at 400 MHz (9.4 T) as described.^4^ Coefficients of variation for intra‐ and inter‐assay precision are <4.3% and <5.5%, respectively. Betaine as measured by NMR is comparable to results obtained by mass spectrometry (*R* ^2^ = 0.94). Crude and multivariable Cox proportional hazards regression analyses were performed to determine the association between plasma betaine levels and microvascular complications. Betaine was log‐base 2 transformed to allow for the expression of the hazard ratios (HRs) per doubling of betaine. Approval for the protocol and informed consent procedure was given by the local ethics committee of Isala hospital, Zwolle, the Netherlands (METC reference numbers 03.0316 and 07.0335).

Betaine levels were determined in 281 participants in the ZODIAC cohort with available data regarding microvascular complications at baseline, of which 139 participants already had microvascular complication(s) at baseline. These and four participants with missing follow‐up data were excluded, leaving 138 participants for the present longitudinal analyses. After a median follow‐up of 3.2 years (range 2.7–3.7 years), 46 out of the 139 included individuals developed one or more microvascular complications. Age at the time of inclusion was 63 (±11) years, 56% was female, average HbA1c levels were 6.9 (±1.2)% (52 [±13] mmol/mol), and median plasma betaine levels were 28 [23–34] μmol/L. Higher values of betaine were associated with a lower risk of developing one or more microvascular complications, as well as with retinopathy and nephropathy as individual outcomes (Table 1 and Figure 1). These associations remained independent of adjustment for age, sex, diabetes duration, smoking, macrovascular complications, HbA1c, total cholesterol, triglycerides and eGFR. In a model adjusted for age, sex, diabetes duration, smoking and macrovascular complications at baseline, a doubling of plasma betaine was associated with a lower risk for developing microvascular complications (HR 0.71 (0.59–0.87)). Results were similar for the development of retinopathy or nephropathy separately.

**TABLE 1:** Associations between plasma betaine and microvascular complications in individuals with type 2 diabetes

	Microvascular complications combined, *Continuous (* ^*2*^ *log)*	*p*	Retinopathy, *Continuous (* ^*2*^ *log)*	*p*	Nephropathy, *Continuous (* ^*2*^ *log)*	*p*	Neuropathy, *Continuous (* ^*2*^ *log)*	*p*
*n*	138		138		138		138	
Events	46		10		16		29	
Model 1	**0.72 (0.69–0.87)**	**<0.001**	**0.62 (0.43–0.89)**	**0.01**	**0.61 (0.46–0.82)**	**<0.001**	1.00 (0.65–1.70)	0.89
Model 2	**0.71 (0.59–0.87)**	**<0.001**	**0.58 (0.38–0.89)**	**0.01**	**0.63 (0.47–0.84)**	**0.01**	1.05 (0.63–1.80)	0.86
Model 3	**0.77 (0.62–0.96)**	**0.02**	0.67 (0.41–1.10)	0.11	**0.66 (0.48–0.91)**	**0.01**	1.18 (0.69–2.00)	0.55
Model 4	**0.75 (0.61–0.91)**	**0.01**	0.71 (0.47–1.10)	0.11	**0.62 (0.46–0.84)**	**0.01**	1.10 (0.65–1.70)	0.68

*Note*: Model 1: adjusted for age and sex.

Model 2: Model 1 + diabetes duration, smoking and macrovascular complications.

Model 3: Model 1 + HbA1c + total cholesterol + triglycerides.

Model 4: Model 1 + eGFR.

Data are presented as hazard ratio (HR) plus 95% CI. *p* Values of <0.05 were considered as significant and are presented in bold.

*HbA1c—*glycated haemoglobin, *eGFR*—estimated glomerular filtration rate.

**FIGURE 1:**
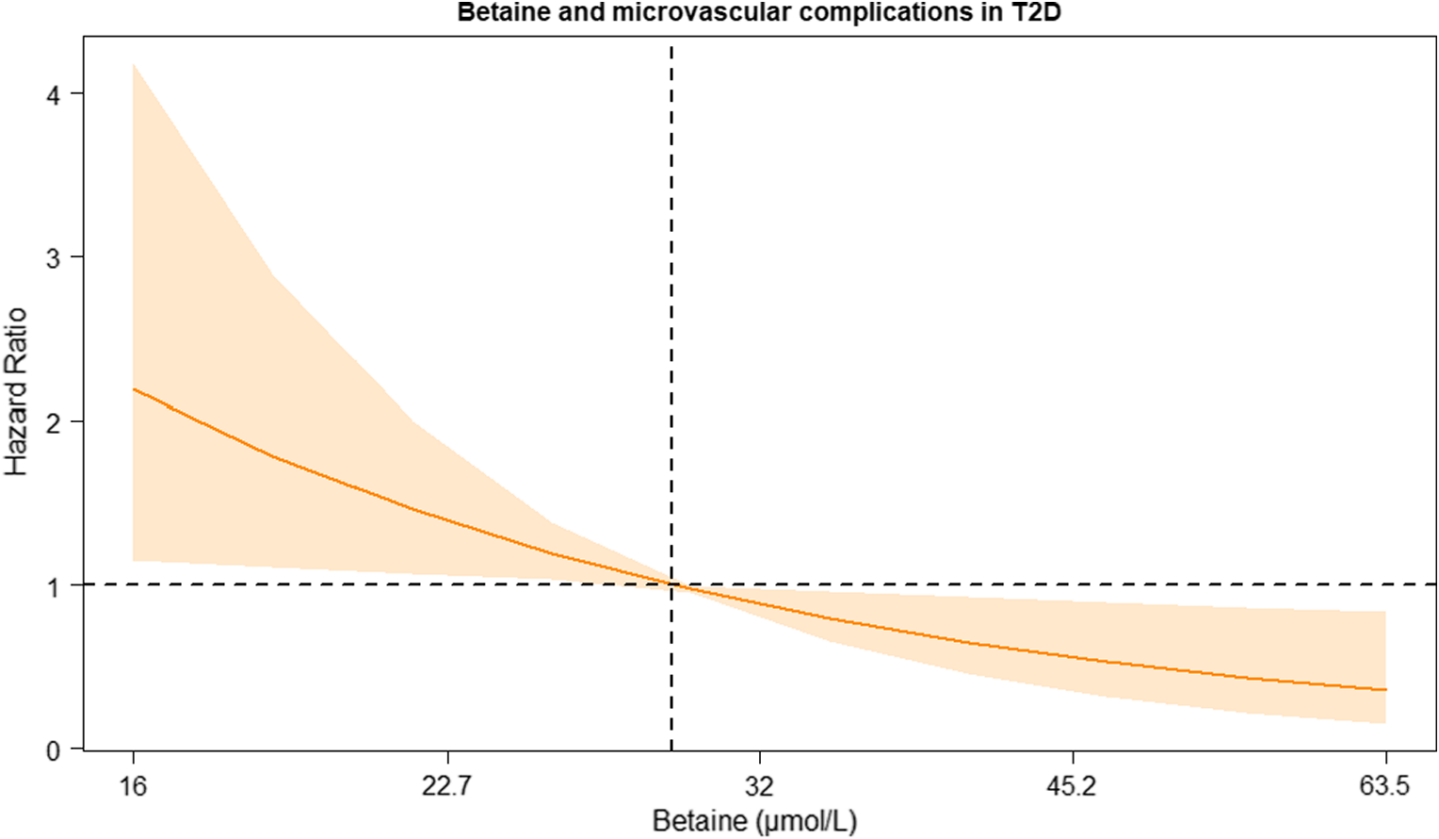
Betaine and total microvascular complications (nephropathy, neuropathy and retinopathy) in individuals with type 2 diabetes. The hazard ratio is shown as a solid line, and the associated pointwise 95% CIs are represented by the shaded area. The depicted hazard ratio is adjusted for age, sex, HbA1c, diabetes duration, smoking, total cholesterol, triglycerides and eGFR.

These longitudinal findings in a contemporary cohort of T2D patients support the hypothesis that plasma betaine is inversely associated with the development of microvascular complications, in particular retinopathy and nephropathy. As our data are observational, no firm conclusions can be drawn regarding causality. Our findings set the stage for future studies addressing whether oral betaine supplementation can prevent microvascular complications in T2D.

## CONFLICT OF INTEREST

The authors have no conflicts of interest to disclose.
